# Tuning the size, composition and structure of Au and Co_50_Au_50_ nanoparticles by high-power impulse magnetron sputtering in gas-phase synthesis

**DOI:** 10.1088/1361-6528/aaf1fa

**Published:** 2019-02-08

**Authors:** A Mayoral, L Martínez, J M García-Martín, I Fernández-Martínez, M García-Hernández, B Galiana, C Ballesteros, Y Huttel

**Affiliations:** 1 School of Physical Science and Technology, ShanghaiTech University, 393 Middle Huaxia Road, Pudong, Shanghai, 201210, People’s Republic of China; 2 Laboratorio de Microscopias Avanzadas (LMA), Instituto de Nanociencia de Aragon (INA), Universidad de Zaragoza, c/Mariano Esquillor, Edificio I + D, E-50018 Zaragoza, Spain; 3 Materials Science Factory, Instituto de Ciencia de Materiales de Madrid, Consejo Superior de Investigaciones Científicas (CSIC), c/Sor Juana Inés de la Cruz, 3, E-28049 Madrid, Spain; 4 IMN-Instituto de Micro y Nanotecnología (CNM-CSIC), c/Isaac Newton, 8, E-28760 Tres Cantos, Spain; 5 Nano4Energy SLNE, Escuela Técnica Superior de Ingenieros Industriales (ETSII-UPM), Instituto de Fusión Nuclear, c/José Gutiérrez Abascal 2, E-28006 Madrid, Spain; 6 Universidad Carlos III de Madrid, Departamento de Física, Av. Universidad 30, E-28911 Leganés, Madrid, Spain

**Keywords:** nanoparticles, gas-phase synthesis, HiPIMS, STEM

## Abstract

Gas-phase synthesis of nanoparticles with different structural and chemical distribution is reported using a circular magnetron sputtering in an ion cluster source by applying high-power impulses. The influence of the pulse characteristics on the final deposit was evaluated on Au nanoparticles. The results have been compared with the more common direct current approach. In addition, it is shown for the first time that high-power impulses in magnetron based gas aggregation sources allows the growth of binary nanoparticles, CoAu in this case, with a variety of crystalline and chemical arrangements which are analyzed at the atomic level.

## 1 Introduction

The increasing demand of pristine nanoparticles (NPs) in nanotechnology is driving a strong effort to find new methods and technologies for the synthesis of NPs. Although the wet chemistry methods are the most popular due to their relative low cost and mass production capacities, the gas-phase synthesis of NPs is getting more attention thanks to its specific advantages. Most of the physical methods are based on the evaporation of a given material followed by a controlled aggregation of the produced ions and neutrals to form clusters or NPs. Since the whole process can be performed under a well-controlled environment (ultra-high vacuum, UHV), the fabricated NPs have the same chemical composition as the evaporated base material, hence avoiding contaminants; also the narrow size distributions can be tuned and improved by the use of filtering techniques [1–11]. Furthermore, this method produces NPs that are free of ligands or surfactants that can be directly deposited on surfaces or embedded in matrices under ultraclean high vacuum environment. Since the seminal work of Haberland’s group [12, 13], magnetron based ion cluster sources (ICS), also known as Gas Aggregation Sources have gained in popularity, essentially due to the fact that they are commercially available, relatively easy to use and that most of the produced NPs are charged, which allows their electrostatic manipulation for deflection of mass filtering for example. Several modifications of the original design have been reported in the literature including the use of hollow cathodes [14–20] and multiple cathodes [21–25]. They are mainly used in straight direct current (DC) and radio frequency (RF) modes, i.e. by applying a constant voltage (current) in DC or an alternative voltage (current) in RF. More limited are the studies were pulsed [26–29] and high-power impulse modes (high-power impulse magnetron sputtering—HiPIMS) [30, 31] have been used to produce NPs. DC, RF and HiPIMS mainly differ in the characteristics of the pulses that in turn have an influence on the degree of ionization of the sputtered material [32]. While DC operation is performed applying a constant voltage to the magnetron, HiPIMS uses high-power densities of the order of Kw cm^−2^ in short pulses of tens of microseconds at low duty cycles (of <10%) [11]. Distinguishing features of HiPIMS are a higher plasma density and thus, an enhanced degree of ionization of the sputtered metal, which results, in combination with a substrate bias, to an increased ion assistance during the coating deposition [33]; the sputtered material ionization increases according to the peak cathode current; the current limit is determined by the transition of the discharge from glow to arc phase while the peak power and the duty cycle are selected so as to maintain an average cathode power similar to conventional sputtering (1–10 W cm^−2^) in order to avoid sputtering target overheating. Anders *et al* defined HiPIMS as: ‘pulsed magnetron sputtering, where the peak power exceeds the time-averaged power by typically two orders of magnitude’ [34]. The fact that the growth rates for thin films are more difficult to control in comparison to the DC mode [35] can explain the limited number of studies of NPs synthesis using HiPIMS. So far, the pulsed mode has been used to prepare TiO*_x_*, Cu [20, 26–28] and Ag [29] clusters and the HiPIMS mode for the growth of Cu and Ag ones [16, 17, 19, 29, 31]. In all these studies, pure single element sputtering targets were used and studies using alloyed targets, to the best of our knowledge, have not been reported so far. Furthermore, there is even a more limited number of studies that have reported the HiPIMS operation of a planar magnetron in a cluster source [30, 31]. These works are focused in silver nanocluster fabrication, which represents a significant smaller size (less than 100 atoms) than the one reported here (up to 24 nm). In addition they used specially designed equipment while in our work, both, the ICS and the HiPIMS power supply are commercially available, which widens the scope of the study.

Here, we report the influence of the applied pulse (average power and frequency) on the mean size, size distribution and deposition rate of pure gold NPs. The study is focused on relatively low average powers and frequencies as compared to already published works with other elements. Secondly, we also investigate for the first time the use of HiPIMS using an alloyed target and evaluate its effect on the structure of bimetallic CoAu NPs. We show that the NPs generated from alloyed magnetron target using a HiPIMS pulse are very different from those generated by DC and present a rich variety of crystalline and chemical arrangements at atomic level.

## 2 Experimental section

### 2.1 Synthesis of NPs

**Figure 1:**
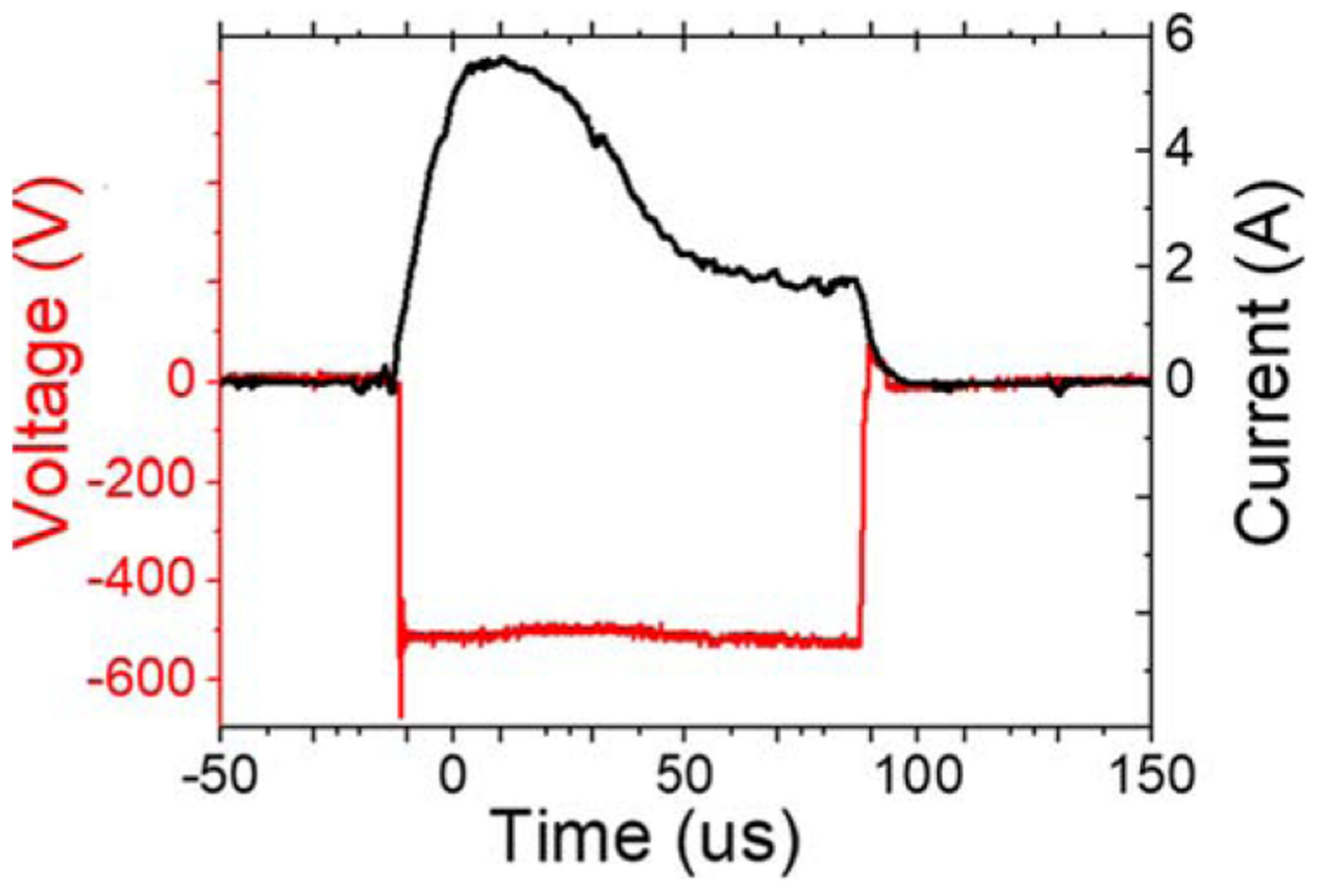
Representative temporal shape of the HiPIMS pulse. The voltage is in red and the current is in black.

The NPs were grown using a single magnetron ICS acquired from Oxford Applied Research Ltd. and connected to an UHV deposition chamber. Both systems have a base pressure of 5 × 10^−10^ mbar. The magnetron was loaded with two inches diameter targets of either pure gold (99.99%) or Co_50_Au_50_ (99.99%). In the present case no mass filtering was applied in order to collect all NPs independently of their mass or charge and no bias was applied to the substrate to increase the NP flux. The DC (LP600) and HiPIMS (hipV 6 kW) power supplies were acquired from Glassman and hipV AB, respectively. Figure 1 displays a representative pulse shape in HIPIMS mode, where the voltage is colored in red and the current appears in black. This pulse has been recorded for an instant power of 2800 W, an averaged power of 38 W and at a frequency of 250 Hz, which corresponds to a duty cycle of 2.5%. The frequency of the pulse, its duration and the intensity maximum have been varied for the different experiments.

For pure Au NPs, both, the average power applied to the magnetron (at fixed frequency of 250 Hz, duty cycle 2.5%) and the frequency (at fixed power of 20 W) were tuned. The real average power was calculated by integrating the voltage and current pulse shapes. For comparison purposes, NPs grown by DC mode were produced at a power of 45 W. All Au NPs were grown with an argon flux of 20 sccm (standard cubic centimetres per minute) and no helium was injected. The magnetron was positioned at an intermediate position inside the aggregation zone, A.Z. (distance from target to exit slit of A.Z. = 45 mm).

CoAu NPs were generated from a Co_50_Au_50_ target. For both, HiPIMS and DC operation modes, the Ar flow was fixed to 45 sccm and the aggregation length was fixed to the maximum length (distance from target to exit slit of aggregation zone = 95 mm). No helium was injected into the aggregation zone. In HiPIMS, the parameters were: average power = 55 W, current peak = 5.1 A, voltage peak = 585 V, duration of pulse = 150 *μ*s, frequency = 250 Hz. Meanwhile, in DC mode the parameters used were: *P* = 98.75 W (*U* = 395 V, *I* = 0.25 A). The values of the parameters in DC were chosen in order to obtain NPs with diameters comparable to those obtained in HiPIMS mode.

The NPs were deposited onto Si(100) polished wafers (roughness below 1 nm) and carbon coated transmission electron microscopy (TEM) grids for their characterization by atomic force microscopy (AFM) and TEM, respectively. In order to avoid charging effects during NPs deposition, p-doped (Boron) Si(100) wafers were used (1.0–10.0 Ohm cm).

### 2.2 Characterization

AFM measurements were carried out using the Cervantes AFM System from Nanotec Electronica S.L. The samples were measured in dynamic mode using tips from Next-Tip S.L. [36] and the images were processed using the WSxM software [37]. TEM measurements were performed with a FEI-TITAN X-FEG TEM used in scanning mode, STEM, mode and operated at 300 kV and at 120 kV. The images were acquired using a high angle annular dark field (HAADF) detector. The microscope was equipped with a monochromator, Gatan Energy Filter Tridiem 866 ERS, a spherical aberration corrector (CEOS) for the electron probe that allows for an effective 0.08 nm spatial resolution, and an energy dispersive x-ray detector for EDS analysis. The magnetic measurements have been performed in a superconducting quantum interference device magnetometer from Quantum Design and equipped with a 5 T (50 kOe) coil. The magnetic field was applied parallel to the sample surface, and the samples were carefully demagnetized before measurements. In the case of field-cooled (FC) measurements the samples were cooled under a magnetic field of 1.5 kOe. The diamagnetic contribution coming from the Si substrates has been carefully measured for further subtraction from the measured data.

## 3 Results and discussion

The first experiments for testing the use of HiPIMS on planar magnetron for the fabrication of NPs were carried out with a single element target. Au NPs were fabricated using different experimental conditions. In particular, two sets of experiments were performed to evaluate the kind of deposits that could be obtained: one as a function of the average power applied to the magnetron (at fixed frequency of 250 Hz, duty cycle 2.5%) and another as a function of the frequency of the applied pulse (at fixed power of 20 W).

**Figure 2:**
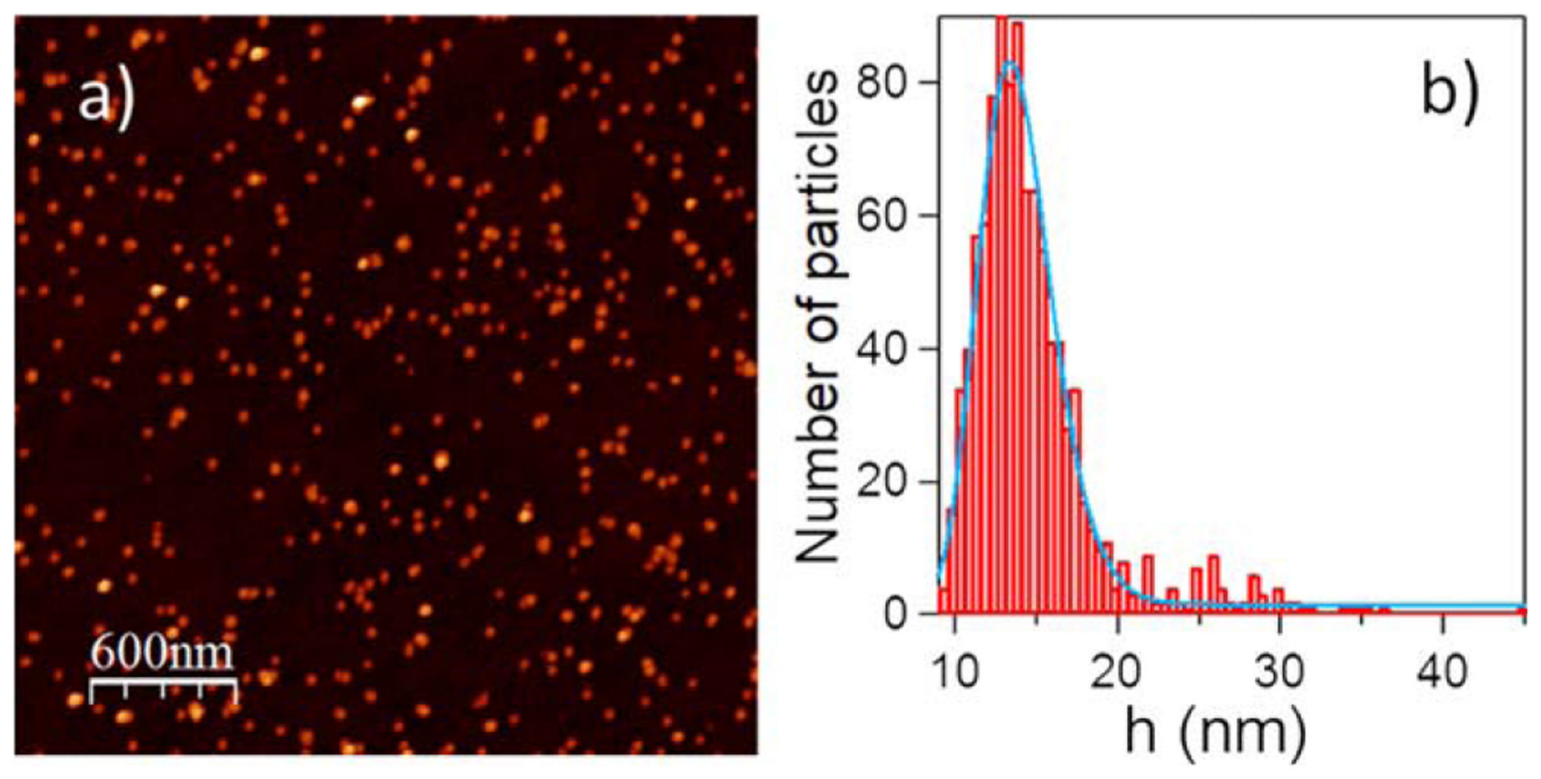
(a) Representative AFM image of Au NPs. (b) NP height distribution extracted from (a).

**Figure 3:**
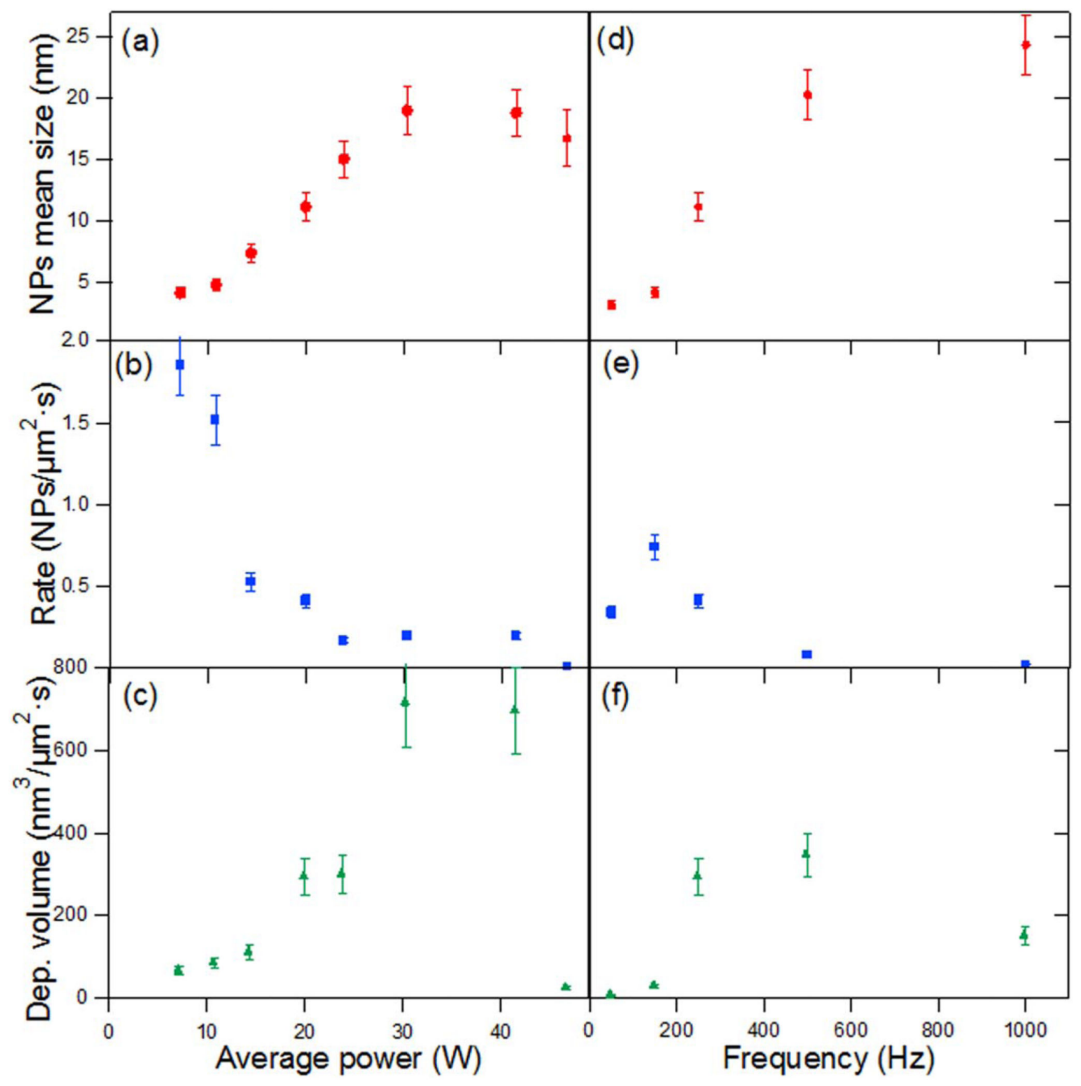
Au NP (a) mean size, (b) deposition rate and (c) volumetric deposition rate as a function of the average power for fixed frequency pulse of 250 Hz. Au NP (d) mean size, (e) deposition rate and (f) volumetric deposition rate as a function of frequency for fixed average power of 20 W.

For each fabricated sample, which corresponds to specific deposition parameters, several AFM images as the one displayed in figure 2(a) were recorded and analyzed. The resulting height distribution of the NPs (see figure 2(b)) has been fitted using a log-normal function in order to extract the mean height and the standard deviation of the size distribution of the NPs. The height of the NPs is assumed to be equal to the diameter as both dimensions were found to be the same in previous studies [38]. This is due to the soft-landing of the NPs that does not induce deformations. The values extracted from the analysis of the samples using different experimental conditions are summarized in figure 3 in terms of NP size, rate and deposited volume. The error bars correspond to the confidence interval extracted from the analysis of different measurements carried out in the samples. For a given frequency of 250 Hz (left column), the increasing average power applied results in an increase of the NP size until 30 W, where this tendency ceased (figure 3(a)). This increase in the NPs mean size with increasing power is in agreement with Zhang’s study performed on Ag nanoclusters [30]. The NP rate, in terms of NPs *μ*m^2^ s^−1^, displayed in figure 3(b) presents the inverse trend. Such tendency would suggest that the sputtered material aggregates into larger NPs more efficiently for higher average powers. The evolution of the deposited volume of NPs (given by the product of the deposition rate and the mean volume) is represented in figure 3(c). It appears that the deposited volume increases almost linearly with the average power up to a maximum at 30 W (the same maximum as in figure 3(a)) and then drops rapidly.

In the case of DC sputtering, it was found that, for an applied power of 45 W, the NPs have a mean diameter of 5.0 ± 1 nm and the deposition rate is of 40 NPs ^−1^ *μ*m^−2^ s^−1^, which represents smaller NP size and significantly higher deposition rate in comparison to HiPIMS. Interestingly, the differences observed between DC and HiPIMS operation modes are in contrast to those observed for the generation of Ag nanoclusters reported in [31], where the authors observed a higher NPs flux when using the HiPIMS mode (approximately nine times higher than DC mode). The different experimental conditions of both studies make difficult a direct comparison. However, it must be taken into account that in Tsuyonama’s study they measured the flux by deflecting the ions and measuring the current using a Faraday cup. It is well known that HiPIMS produces a larger degree of ionization. Therefore, as they explained in their work, this could be the cause for the enhanced detection in HiPIMS in comparison to DC. In our case, no filtering was applied and neutral and charged NPs are collected on the substrate. Therefore, this difference in the ionization process is not as evident as if we only analyzed the charged NPs.

Figures 3(d)–(f) presents the evolution of the same parameters as figures 3(a), (c), although in this case as a function of the frequency at a fixed *P* = 20 W. There is a clear dependency of the NP mean size on the frequency (figure 3(d)). It increased by a factor of 10 with the increasing frequency of the pulse. This behavior is in agreement with a previous study performed on the growth of Ag nanoclusters [30] although the experimental parameters and NPs sizes strongly differ, therefore suggesting that such trend with increasing frequency is probably general. In addition, the deposition rate rapidly dropped at higher frequencies (figure 3(e)). However, since the NPs size is bigger at high frequencies, the deposited volume per unit surface and time is bigger at frequencies higher than 200 Hz (figure 3(f)). From the data presented in figure 3 it is possible to extract that the NP size can be tuned from 2 to 24 nm in the power and frequency range presented here by operating the cluster source using HiPIMS. However, it has to be taken into account that the optimal parameters to obtain big NP sizes are clearly associated to a significant decrease in the deposition rate.

### 3.1 Co_50_Au_50_ NPs

Once that the ability to tune the NP fabrication process was proved using HiPIMS in a planar magnetron, the next step of this work was to extend this procedure for the fabrication, for the first time, of more complex NPs using this HiPIMS method, and evaluate whether the fabrication procedure has an influence on the structure of the NPs. With this aim, NPs fabricated from a Co_50_Au_50_ target in DC and HiPIMS were deposited at room temperature (RT) on carbon coated TEM grids for comparison purposes. The chemical composition and structure of the NPs was estimated by spherical aberration corrected (C_s_-corrected) STEM-HAADF imaging coupled with spectroscopic measurements.

**Figure 4:**
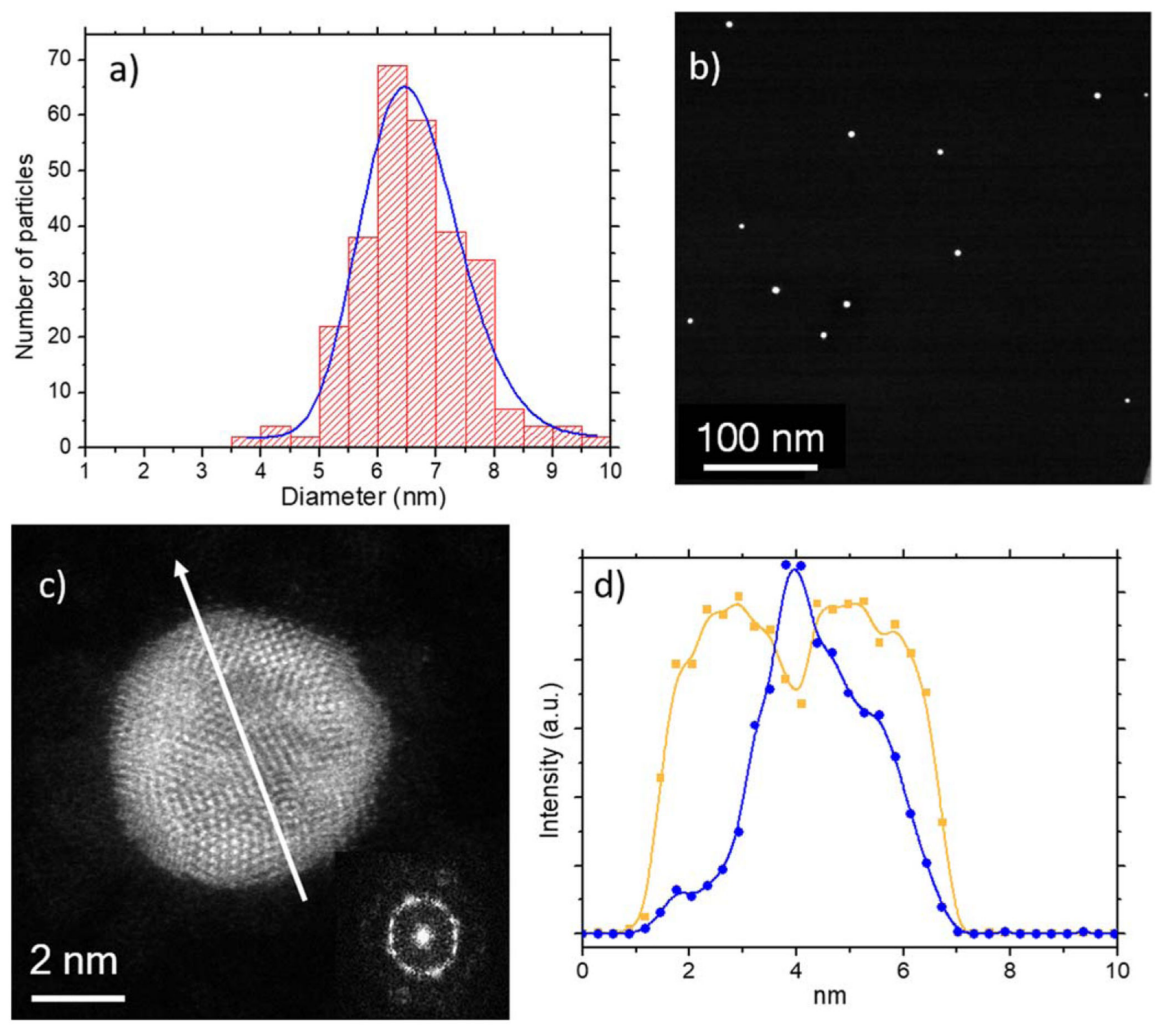
(a) Particle size distribution of Co_50_Au_50_-DC at room temperature. (b) Low-magnification C_s_-corrected STEM-HAADF image of Co_50_Au_50_-DC, showing several NPs. (c) C_s_-corrected STEM-HAADF image of Co_50_Au_50_-DC; the white arrow represents where the EDS profile was extracted from. (d) EDS profiles Co in blue and Au in dark yellow clearly showing that the Au rich zone is in the shell of the NP.

**Figure 5:**
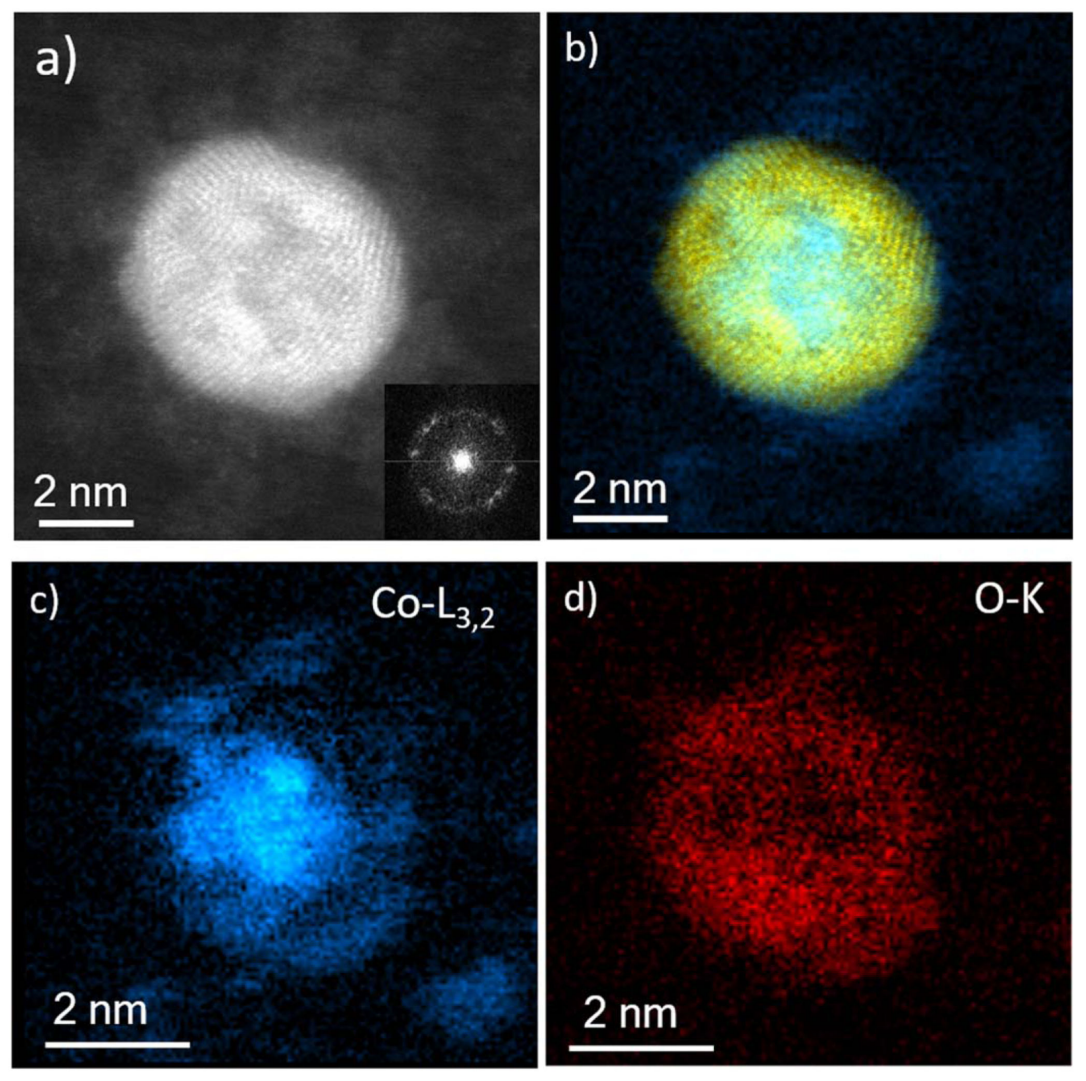
C_s_-corrected STEM-HAADF images of a representative NP generated by DC mode at RT. (a) Z contrast image where the multi-twined structure can be observed, the extracted FFT is shown inset, revealing the polycrystalline nature of the NP. (b) HRSTEM image colored based on its composition where Au is in yellow and Co in blue. (c), (d) Represent the spectrum imaging images corresponding to Co-L_3,2_ signal in blue and the O–K signal in red.

The electron microscopy statistical studies performed on 286 NPs fabricated using DC revealed the formation of particles with a homogenous size distribution with average diameter of 6.62 ± 0.98 nm (see figure 4(a)). Low-magnification images illustrate such homogeneous size distribution and confirm the absence of smaller or larger NPs (see figure 4(b)). The chemical composition of the NPs was estimated by spherical aberration corrected (C_s_-corrected) STEM imaging coupled with spectroscopic measurements. As can be observed in figure 4(c), core@shell NPs are spontaneously formed by using DC. Unlike in other core@shell Co@Au NPs [39, 40], the crystalline structure of these Co_50_Au_50_ NPs can be described as multi-twined NPs (as can be corroborated by the fast Fourier transform (FFT), inset of figure 4(c) composed by a cobalt rich core which is not pure as some mixture with gold can be clearly visualized in terms of Z contrast. An EDS profile performed along the NP, denoted by the white arrow of figure 4(c) is depicted in figure 4(d), where the core@shell structure can be clearly visualized. A deeper chemical analysis of the NPs was performed by means of electron energy loss spectroscopy. Figure 5(a) corresponds to the NP in which a spectrum image analysis was performed; the Co-L_3,2_ signal (779 eV) is represented in blue color in figure 5(b) while the strongest signal that corresponds to Au is represented in yellow. In addition to the Co edge (figure 5(c)), the O–K edge could be also identified on the surface of the NP (figure 5(d)). As can be seen in the chemical maps, certain amount of cobalt was also present at the surface of the NPs forming a fine layer of disordered cobalt oxide covering, at least partially, each NP. This cobalt oxide appeared as a consequence of the interaction of cobalt with the oxygen from air exposure.

**Figure 6:**
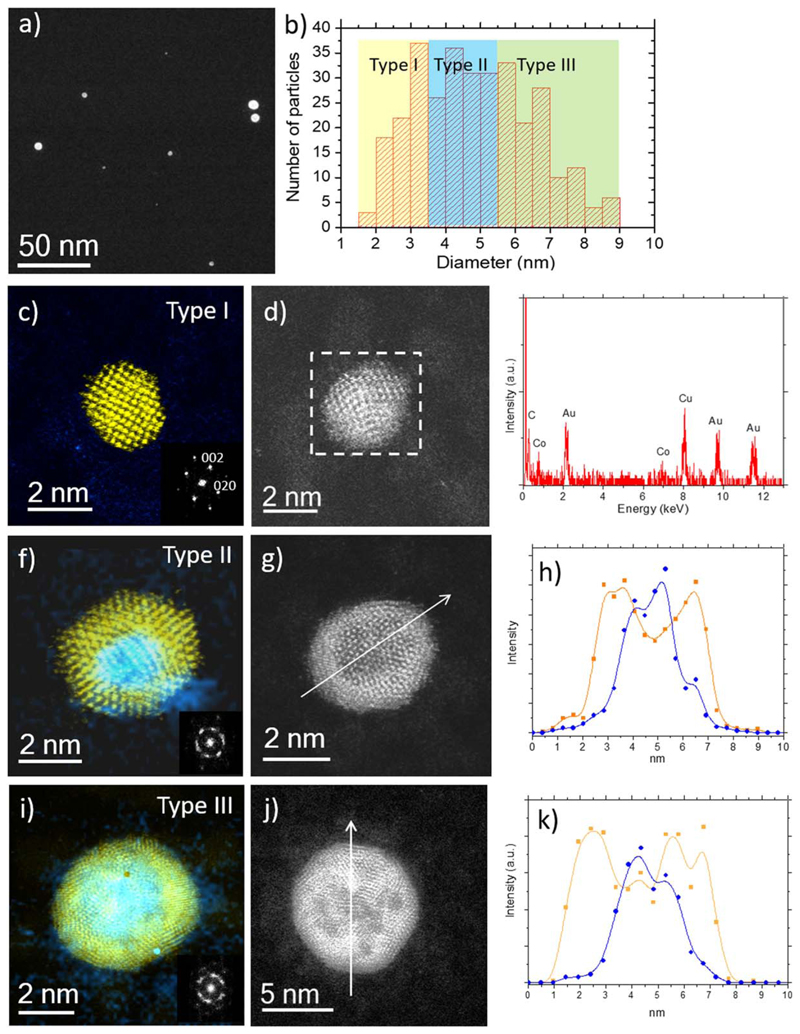
Analysis of the Co_50_Au_50_ HiPIMS at room temperature. (a) Low-magnification C_s_-corrected STEM-HAADF image showing the different types of NPs. (b) Particle size distribution. Type I: (c) Au NPs partially covered by Co atoms. (d) Pure Au fcc NP and (e) the corresponding EDS analysis, where the Co signal is associated to the cobalt oxide located at the surface of the NP; due to the lower content in oxygen, EDS signal was not sufficient for performing the line spectroscopic analysis. Type II (f) core@shell Co@Au NPs. (g) 5 nm Co_50_Au_50_ NP with icosahedral morphology together with (h) chemical composition along the white arrow. The blue corresponds to the cobalt signal while the dark yellow corresponds to the gold signal. Type III (i) NPs with a rich Co core partially mixed with Au and an Au shell with adsorbed cobalt oxide at the surface. (j) C_s_-corrected STEM-HAADF image of a polycrystalline NP where the chemical composition (k) was analyzed along the white arrow; the blue corresponds to the cobalt signal while the dark yellow corresponds to the gold signal.

The same electron microscopy analysis was carried out on NPs generated by HiPIMS and deposited at RT (figure 6). Low-magnification image (figure 6(a)) evidences a less homogeneous size distribution than DC sample. Thus, the results of the particle size analysis performed on 320 NPs (figure 6(b)) differ significantly from the DC Co_50_Au_50_ NPs as the NP size ranges from 2 nm up to 9 nm in a broad size distribution. Interestingly, inside this broad size distribution, three different types can be distinguished of NPs according to their chemical compositions and structures. Type I corresponds to the smallest NPs (below 4 nm), with a mean diameter of 2.96 ± 0.52 nm. They are mainly composed by pure crystalline gold with face-centered cubic (fcc) structure with cobalt surrounding the NPs. These NPs represent 25% of the total. Figure 6(c) shows an Au single crystal NP observed along the [100] beam orientation. The well-defined diffraction spots observed in the FFT corroborate the single crystal nature exempt of structural defects. The spectroscopic measurements performed in one of these NPs (figure 6(d)) revealed that the composition of the smallest NPs was predominantly pure Au, with a very small amount of Co which was associated with the presence of this element on the surface in the form of cobalt oxide (figure 6(e)). Type II NPs corresponds to NPs with mean diameter of 4.76 ± 0.42 nm. They are composed by a pure Co core surrounded by an Au shell forming a core@shell Co@Au structure that was also partially covered by cobalt oxide. Such NPs, represent 37% of the total. Figure 6(f) presents one of these NPs, a 4.30 nm diameter icosahedron sitting along its two-fold axis. EDS analysis (figure 6(h)) of the NP shown in figure 6(g) evidenced a significant increase in the Co signal in comparison to the analysis of type I NPs. Finally, type III NPs corresponded to NPs larger than 5 nm, with mean diameter 6.70 ± 0.82 nm. They exhibited the same morphology as the NPs generated by DC mode (figure 6(i)) i.e. a rich Co core, but partially mixed with Au and an Au shell with adsorbed cobalt oxide on the surface. These NPs represent 38% of the deposited NPs. A line profile was also performed in one of these NPs (shown in figure 6(j)) where Co appears in blue and Au in yellow proving that the darker contrast corresponds to Co while the brightest areas are associated to Au corroborating the correlation between the image contrast observed and the spectroscopic measurements displayed in figure 6. The histogram displayed in figure 6(b) illustrates the distribution of the different populations of NPs. The difference in size of each type of NP fabricated by HiPIMS would allow, by using a quadrupole mass filter, the selection one particular structure if needed.

**Figure 7:**
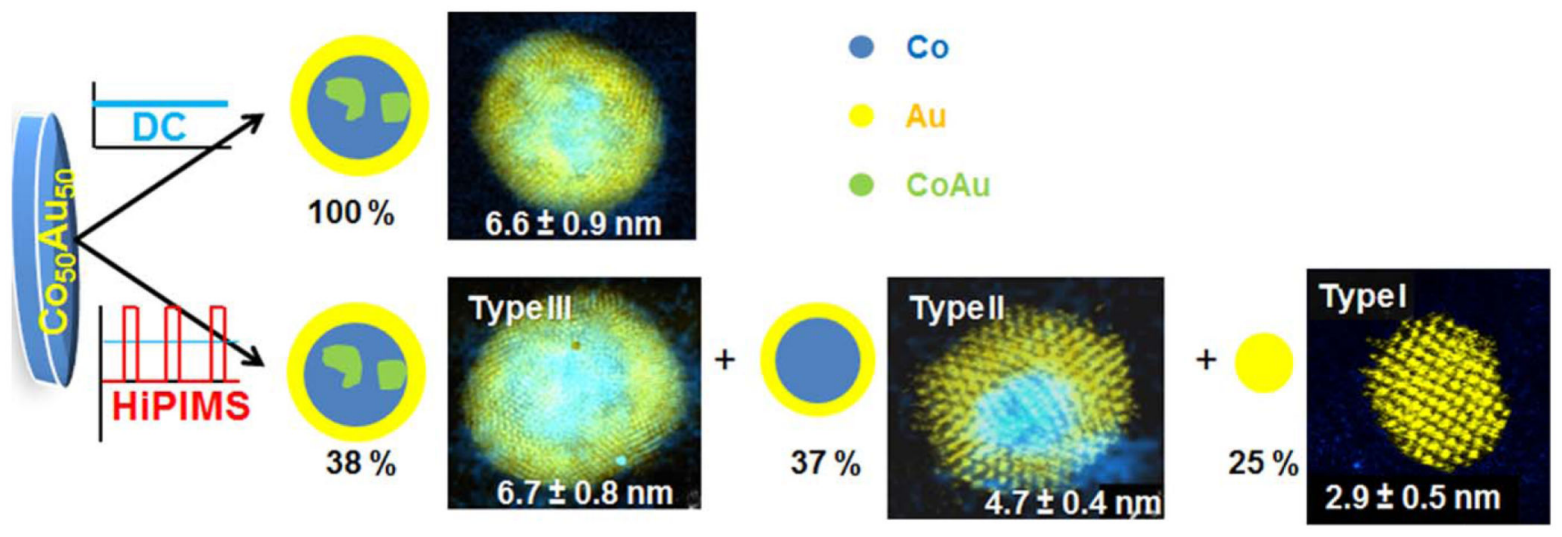
Schematic representation of the NPs structure obtained from the same target using DC (top) or HiPIMS (bottom). The average size and abundance of each kind of NP is detailed in each image.

Therefore, from this study of the CoAu system it is evidenced that the use of HiPIMS on binary systems leads to the formation of NPs with structures different from the ones obtained by DC sputtering. While the use of DC results in the formation of only one type of core@shell NP, homogeneous in size and structure, the use of HiPIMS results in a new phenomenology where a tri-modal distribution, each corresponding of a different kind of NP, was found. Figure 7 presents a schematic representation of the structures obtained using DC and HiPIMS.

HiPIMS sputtering is a more energetic process than conventional DC sputtering. This excess of energy could be responsible for the formation of the wider variety of NP structures found in comparison to DC sputtering. It is well known that HiPIMS produces a higher plasma density and thus, an enhanced degree of ionization of the sputtered metal [33]. As a consequence, there is a larger proportion of ions in comparison to DC mode. As a general trend, ions are very effective condensation germs [11] and one would expect a larger proportion of aggregates in these conditions. The high energetic pulses seem to increase the diversity of these condensation germs that lead to different kinds of NPs. In addition, the excess of energy already mentioned could lead to a longer period were the NPs are in an *excited* state while they cool down before reaching a final and stable state. This longer period with an exceeding energy may enable different processes, such as diffusion, to occur. At the nanoscale, the size dependent phenomena could lead to different behavior of the NPs depending on their size. In the case of the NPs fabricated by HiPIMS, we observed a wider size distribution than in DC. As it was previously mentioned, this size distribution was correlated to different NPs structure. The smallest NPs (type I, 2.9 nm) are pure Au NPs arranged in a perfect crystal, where the Co atoms have migrated to the outer part of the particle. At this size, any process would be highly favored by an excess of energy carried by the NP. Furthermore, the smallest NPs will need less excess of energy to promote changes of crystalline structure and to favor diffusion of the elements. The atoms tend to diffuse to positions that lead to a decrease of the surface free energy of the whole NP [41]. This could explain the surface segregation of Co atoms observed in this type of NPs. In the case of the intermediate sized NPs (type II, 4.7 nm), the excess of energy proportioned by HiPIMS mode seems to still enable diffusion process and a pure Co core surrounded by a pure Au shell is obtained. This kind of diffusion of both elements to form a pure core and a pure shell is not observed in NPs with larger size (type III, 6.7 nm), where a mixed CoAu core is still present, as occurred in the case of DC sputtering. The extra energy proportioned by the HiPIMS mode is not enough to promote the same diffusion processes observed in the smaller NPs. Therefore, with the experimental conditions used in this work, the extra energy from the high energetic pulses is enough to promote migration of species in binary NPs with sizes below the threshold size between type II and type III NPs (4.7–6.7 nm). One has to keep in mind that this work has been carried out with Co and Au, two elements that are poorly miscible in the bulk phase. Hence, the tendency to create two separated phases is favored. Previous works carried out in this sense [39, 41] demonstrate that providing temperature to AuCo alloyed NPs induced the migration of Au to create Co core-Au shell NPs. In order to obtain more insights of the NP fabrication using HiPIMS, further investigations are needed, although this work evidenced that HiPIMS provides an alternative route to produce NPs with new structures that cannot be accessible by other approaches without additional treatments.

**Figure 8:**
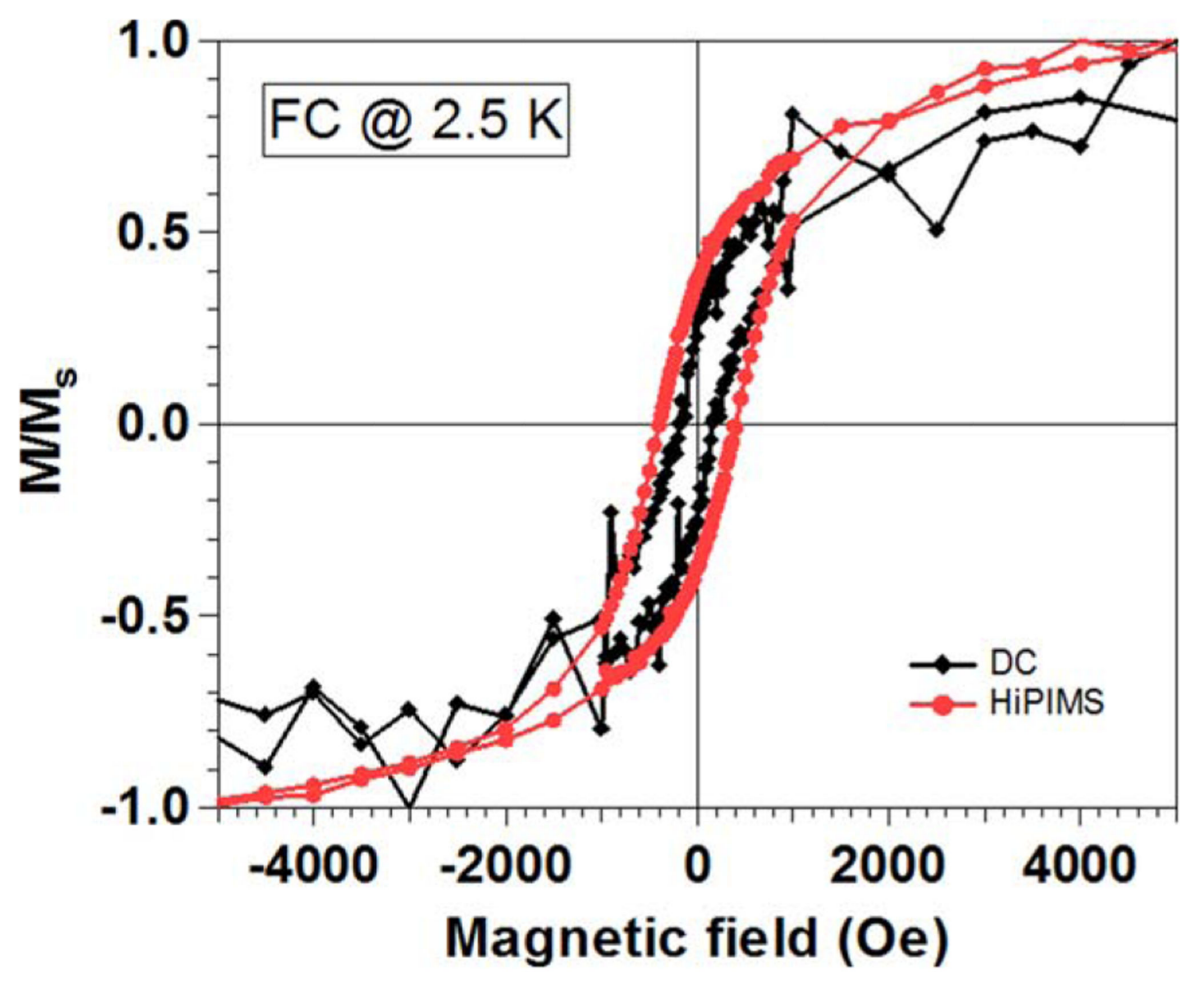
Normalized field-cooled hysteresis loops measured at 2.5 K of Co_50_Au_50_ NPs generated by DC and HiPIMS modes.

Such diversity of sizes and structures has an influence of the final properties of the fabricated systems. As an example of this, figure 8 presents a comparison of the FC hysteresis loops of CoAu NPs fabricated by means of DC and HiPIMS sputtering, normalized to saturation magnetization for easier comparison. It can be observed that the coercivity of the HiPIMs sample is clearly larger than the DC sample. This difference may arise from the fact that with HiPIMS mode, a pure Co core is formed surrounded by a protective Au shell in the 31% of the NPs (type II NPs) and, thus, a stronger magnetic response is expected on this sample [39]. Despite the presence of a thin cobalt oxide shell on the surface of the NPs, we note the absence of a clear exchange bias in the samples. The small volume of this antiferromagnetic cobalt oxide that is surrounding the ferromagnetic core is probably not enough to develop a clear exchange bias effect.

## 4 Conclusions

In this work HiPIMS has been tested in a planar magnetron gas aggregation source as a fabrication method of clean, ligand-free NPs. A detailed study as a function of the pulse parameters (frequency and power) carried out with an Au target revealed that it is possible to tune the NP size from 2 to 24 nm by changing the pulse conditions. The comparison with the deposits on DC mode evidences the possibility to fabricate much bigger NPs, although with a smaller deposition rate.

Binary CoAu NPs have been fabricated for the first time using HiPIMS in a sputter gas aggregation source. From the comparison with DC growth, NPs with new structures are formed by using HiPIMS. While DC leads to the formation of core@shell structures with a CoAu core surrounded by an Au shell, the use of HiPIMS leads to the formation, not only of the same kind on NPs as with DC, but also to pure Co core surrounded by an Au shell NPs with smaller size and also pure Au NPs with even smaller size (below 4 nm). Most NPs, irrespective whether they are fabricated by DC or HiPIMS, present a thin cobalt oxide layer or on their surfaces.
